# How Many Seals Were There? The Global Shelf Loss during the Last Glacial Maximum and Its Effect on the Size and Distribution of Grey Seal Populations

**DOI:** 10.1371/journal.pone.0053000

**Published:** 2012-12-26

**Authors:** Lars Boehme, Dave Thompson, Mike Fedak, Don Bowen, Mike O. Hammill, Garry B. Stenson

**Affiliations:** 1 Sea Mammal Research Unit, Scottish Oceans Institute, University of St Andrews, St Andrews, United Kingdom; 2 Bedford Institute of Oceanography, Department of Fisheries and Oceans, Dartmouth, Nova Scotia, Canada; 3 Department of Fisheries and Oceans, Maurice Lamontagne Institute, Mont Joli, Quebec, Canada; 4 Department of Fisheries and Oceans, Northwest Atlantic Fisheries Centre. St. John's, Newfoundland, Canada; National Oceanic and Atmospheric Administration/National Marine Fisheries Service/Southwest Fisheries Science Center, United States of America

## Abstract

Predicting how marine mammal populations respond to habitat changes will be essential for developing conservation management strategies in the 21st century. Responses to previous environmental change may be informative in the development of predictive models. Here we describe the likely effects of the last ice age on grey seal population size and distribution. We use satellite telemetry data to define grey seal foraging habitat in terms of the temperature and depth ranges exploited by the contemporary populations. We estimate the available extent of such habitat in the North Atlantic at present (between 1.42·10^6^ km^2^ and 2.07·10^6^ km^2^) and at the last glacial maximum (between 4.74·10^4^ km^2^ and 2.11·10^5^ km^2^); taking account of glacial and seasonal sea-ice coverage, estimated reductions of sea-level (123 m) and sea surface temperature hind-casts. Most of the extensive continental shelf waters (North Sea, Baltic Sea and Scotian Shelf), currently supporting >95% of grey seals, were unavailable during the last glacial maximum. A combination of lower sea-level and extensive ice-sheets, massively increased seasonal sea-ice coverage and southerly extent of cold water would have pushed grey seals into areas with no significant shelf waters. The habitat during the last glacial maximum might have been as small as 3% of today's extent and grey seal populations may have fallen to similarly low numbers. An alternative scenario involving a major change to a pelagic or bathy-pelagic foraging niche cannot be discounted. However, hooded seals currently dominate that niche and may have excluded grey seals from such habitat. If as seems likely, the grey seal population fell to very low levels it would have remained low for several thousand years before expanding into current habitats over the past 12,000 years or so.

## Introduction

The greatest challenges facing marine ecologists in the 21st century will be understanding, predicting and where possible, ameliorating the effects of climate change. Marine mammals are upper-trophic level predators in the marine environment and are often cited as indicator species for environmental health [1]. As large, charismatic and often highly visible components of the marine ecosystem, they are of major public interest and their conservation and management are important issues in their own right. Understanding the specific mechanisms by which climate change will affect marine mammals and predicting how their populations respond to habitat changes will be essential for developing conservation management strategies that can help prevent or mitigate any negative impacts [2]. How marine mammals respond to changes in their environment depends on their adaptability, and the temporal and spatial scale of perturbation.

Marine mammals are long lived, wide ranging animals and as a result must be examined on an ecological scale that ranges from years to decades and from tens to thousands of kilometres. These temporal and spatial scales are small compared with evolutionary and geologic scales, but large compared with human research and resource management scales [3]. Although the spatial and temporal scope of today's scientific research and monitoring is increasing, consistent long-term sampling is much harder to achieve, in part because long-term monitoring projects are hard to fund [4]. This mismatch in scales complicates the task of predicting impact and assessing resilience for marine mammals in the face of climate change.

Many marine mammals undertake large-scale seasonal migrations and therefore frequently experience changing environmental conditions [5]. As a result, they are likely to have developed the capacity to tolerate sudden interannual changes. In addition, they have clearly survived repeated periods of cooling or warming over evolutionary time [6]. In some cases, however, long-term unidirectional changes can result in permanent habitat change or even habitat loss, which may have a significant impact on their populations [7]. Nevertheless, even short-term changes of the physical environment can cause changes in marine mammal populations by affecting demographic parameters, e.g. pup survival [8].

Recognition that climate can change animal habitats is not new. Darwin [9] noted that advancing glaciers must have pushed temperate animals southward, while Arctic species took their place and vice versa. Vibe [10] described quantitative impacts of climate change on marine mammals in West Greenland, where multi-decadal environmental fluctuations altered the density and distribution of top predators.

But can we predict future changes in animal habitats? Attention has focused on the impacts of climate change on Arctic and particularly ice associated marine mammal species [2]. The Arctic is projected to warm at about twice the rate of the global average [11], [12]. The summer extent of the Arctic sea ice cover has decreased in recent decades and the timing and duration of the summer melt season have changed [13], [14]. Future scenarios with a continued ice albedo feedback show an accelerated decrease in sea ice cover and thickness and suggest a tipping point leading rapidly to an Arctic Ocean with substantially reduced summer sea ice [14]. Such abrupt changes may exceed the ability of Arctic species to adapt and are a subject of much current research [12]. Some investigations show that up to 37% of species can be ‘committed to extinction’ based on a mid-range climate-warming scenario for 2050 [15]. While there has been an increased focus on the Arctic and its marine mammal fauna, very few studies have examined the likely impacts on marine mammals in temperate seas [16].

The effects of climate change on temperate ecosystems may appear less dramatic, but are nevertheless present [16]. Since, at present our ability to predict such effects on marine mammal populations is limited it may be informative to examine the effects of previous environmental changes to obtain insights into the likely responses of populations to future climate trends [17]. Current conditions that are routinely regard as ‘normal’ are, in fact, the result of a dramatic and rapid warming event following the last glacial maximum (LGM). Approximately 21,000 years ago our planet was experiencing the last full glacial conditions. Vast ice sheets covered much of the land masses in the northern hemisphere and the sea level was reduced by about 123 m, profoundly changing the distribution and availability of shallow continental shelf waters [18]. Lower atmospheric and ocean temperatures, and increased glacial conditions also meant that both winter and permanent summer sea ice extended far south [19]. Clearly these changes would have had profound effects on distribution and perhaps population size for marine mammals living in both polar and temperate regions.

In terms of their biodiversity and productivity, temperate continental shelf waters are extremely important. Although the continental shelf only accounts for about 0.5% of the ocean's volume, recent observations have shown that the annual primary production is about 16% of the global ocean production [20], [21] and supporting major fisheries and large populations of marine mammals and seabirds [22]. In this study, we look at the profound changes in marine habitat on the continental shelf between the LGM and today. One simple proxy is the change of the continental shelf areas caused by the change in sea level, which must have had an enormous impact on the productivity and biodiversity of the oceans. Here, we look at this change and discuss its impact on the continental shelf habitat by focusing on the biology of one well-studied marine mammal species, the grey seal (*Halichoerus grypus*) to illustrate the importance of these habitat changes. First, we define current grey seal habitat by simple environmental proxies using bio-logging information from satellite relay data loggers in conjunction with global environmental SST and bathymetry data. By selecting proxies that are also available from ocean and climate reconstructions of conditions at the LGM we are then able to examine the likely effects of the LGM on grey seal population size and distribution in particular.

## Materials and Methods

### Climatic data

**Figure 1:**
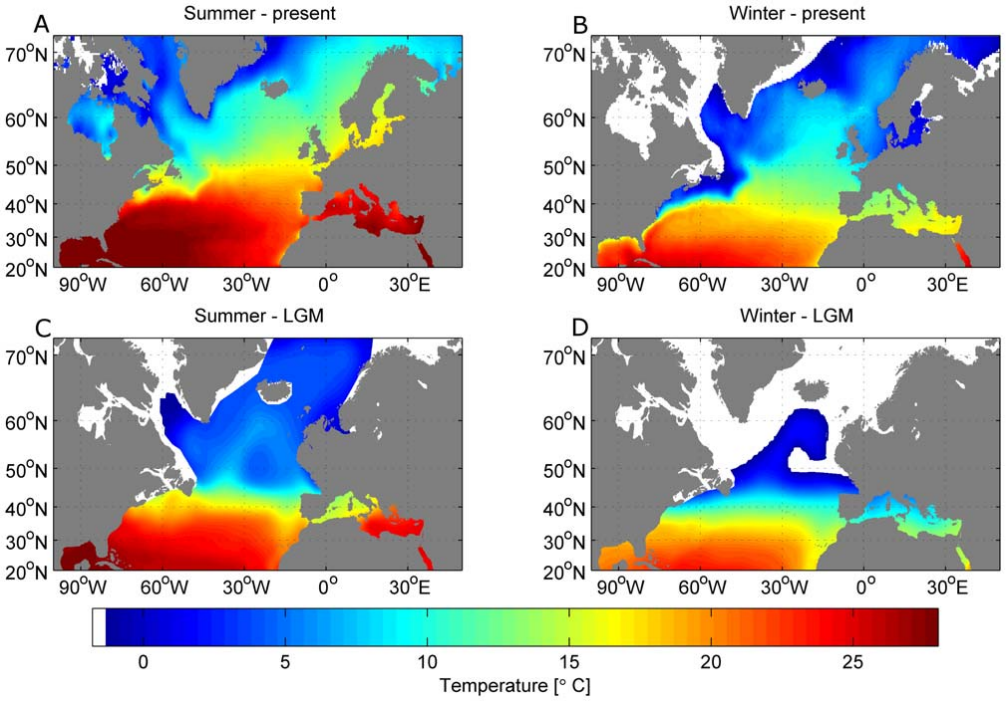
Environmental conditions today and during the LGM. *Top row*: Sea Surface Temperature (SST) for February (A) and August (B) from the gridded SST dataset and corresponding sea ice extend (white) for today. *Bottom row*: SST during the LGM based on GLAMAP data for February (C) and August (D). Land is grey and is based on the 5-minute Terrainbase elevation data, lifted by 123 m for the LGM dataset. Ice sheet and sea ice extent are shaded white.

The only proxies available characterizing the oceans today and during the LGM on the necessary scale are bathymetry and sea surface temperature. To describe current oceanographic conditions, we use the quarter degree sea surface temperature (SST) analysis of the World Ocean Atlas 2005 [23]. A 25 km ×25 km sea ice climatology (1972–2004) was used to produce the winter and summer sea ice extents [24]. To avoid excessive computation time we used a relatively coarse bathymetry based on the 5-minute Terrainbase elevation data [25] to describe the ocean's bathymetry and extrapolated the SST data onto the same 5-minute grid. The present winter (February) and summer (August) conditions for the North Atlantic are shown in figure 1.

The oceanic environmental parameters of the North Atlantic during the LGM are crucial to climate studies and have been studied intensively for many decades [26]–[29]. In this paper, we describe the North Atlantic at the LGM by using gridded monthly sea-surface boundary conditions [30], based on the sea-surface temperature reconstruction of the GLAMAP project [28]. The bathymetry was again based on the 5-minute Terrainbase elevation data. However, sea level was different during the LGM. Results from ice sheet and sea level models identify a range of possible solutions for ice volume, expressed as ice-equivalent sea level lowering, from a minimum of 118 m to a maximum of 135 m [19], [31]. The magnitude of the sea level reduction is still the focus of intense debate [18], but for the purpose of this study, we adopt the results from Hanebuth et al. [18] and define the eustatic sea-level change during the LGM to be 123 m lower than present. Thus, we added 123 m to the elevation data (water depth negative) to obtain the LGM bathymetry. The winter and summer conditions during the LGM are shown in Figure 1.

To calculate the shelf area or habitat extent, we summed the area of all grid cells which fit the environmental conditions (depth and SST) described later and are not covered with permanent glacial ice or sea ice.

### Grey seal data

The movement patterns, at sea distributions and foraging behaviour of grey seals have been extensively investigated using satellite-relay data loggers (SRDLs). These SRDLs collect and process dive information (e.g. dive profile shape and maximum depth) and transmit these data via the Argos satellite system [32]–[34]. Argos also provide position information [35], [36] giving approximately 5 locations per day per seal while they are at sea. To date in excess of 400 grey seals have been tracked using this system [37]–[42].

**Figure 2:**
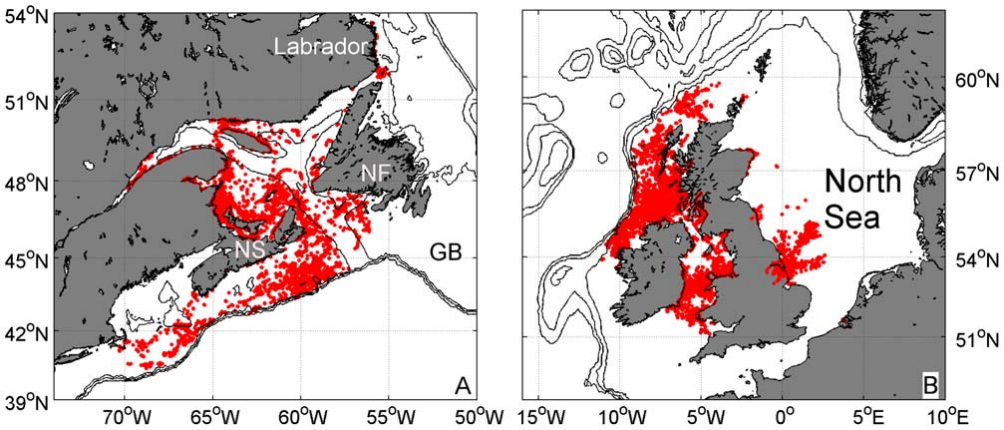
Grey seal telemetry locations. 34,140 grey seal locations (red dots) as recorded by 81 SRDLs between May 2003 and May 2007 with water depths greater than 0 m in the western (A) and eastern (B) North Atlantic. Isobaths are 200 m, 500 m and 1000 m and land is shaded grey. Some important topographic features are marked: Newfoundland (NF), Nova Scotia (NS) and Grand Banks (GB).

To estimate the values of depth and water temperature that define suitable grey seal habitat, we used information from a subset of 81 grey seals that were fitted with SRDLs between May 2003 and May 2007 in the UK and Canada [32], [37], [41], [42]. We selected the sub-sample from the central portion of the latitudinal range of the known breeding distribution on both sides of the Atlantic. We extracted maximum dive depths from 223,157 individual dive records from approximately equal numbers of seals on the east and west side of the North Atlantic Ocean. In addition, for each ARGOS location estimate, we extracted the water depth from the 1 arc-minute elevation data of the ETOPO1 global relief model [43]. ARGOS locations were filtered to remove errors [44] and locations recorded during haul-out periods. We used 34,140 locations, which had a calculated water depth of more than 0 m, indicating that the location was ‘at sea’ (Fig. 2). By using the two depths estimates, we can compare the actual dive depths to the available water depths at that location. Temperature range was determined in two ways. Most SRDLs were equipped with a temperature sensor with an expected accuracy of better than 0.5°C [45], [46]. The deepest temperature-depth profile recorded during each hour was stored within the SRDL, but due to the limited data throughput via the ARGOS satellite system only between 6–12 temperature profiles each day were received [33], [47]. Nevertheless, a total of 54,226 temperature profiles were obtained, which provide an accurate description of the temperature range experienced by seals. In addition, we estimated the annual mean SST for each of the 34,140 ARGOS derived locations using data from the gridded SST data.

## Results

### The global loss of shelf areas

The continental shelf is defined as the shallow and rather flat seafloor between the coast line and the shelf break. The shelf break is usually associated with a steep slope and moving from e.g. the 200 m to the 500 m isobaths involves little horizontal movement. Nevertheless, in some areas (e.g. European Arctic, Norwegian coast and Greenland) the shelf break occurs beyond the 500 m isobath. Thus, for the purpose of this study, we define the continental shelf as an area with a water depth of less than 500 m, which is not covered permanently with sea ice or glacial ice. The resulting total shelf area of today is then estimated to be 3.13·10^7^ km^2^, which is about 9% of the ocean's total area. We then lowered the sea level by 123 m to simulate sea levels during the LGM, resulting in a shelf area of 8.48·10^6^ km^2^, which is about 2.43% of the ocean's total area, a decrease of 73% from the present value. This area change between the LGM and today is entirely the result of a sea level rise and changing extent of permanent ice.

**Figure 3:**
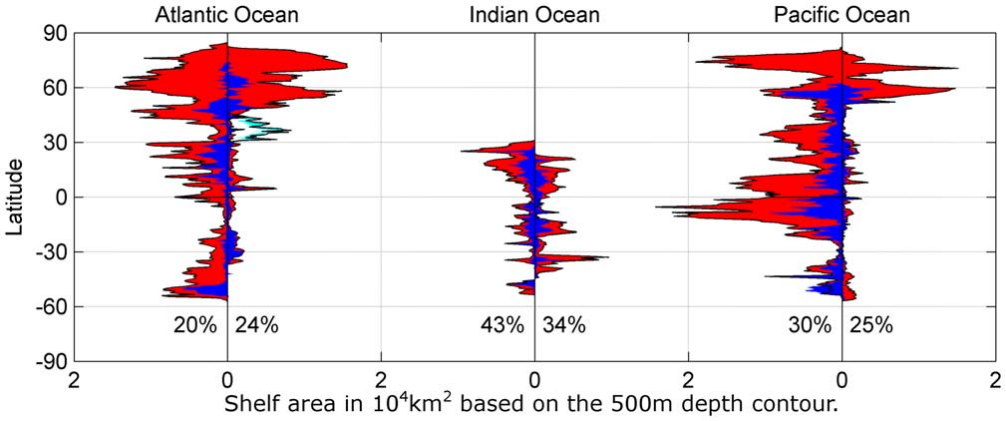
Shelf areas today and during the LGM. Available continental shelf area (<500 m) during the LGM (blue) based on a sea level drop of 123 m and today (red) per degree latitude. The left panel shows the Atlantic Ocean. The western shelf areas are on the left side of the zero line, while the eastern shelf areas are on the right side, excluding the Mediterranean Sea, which is shown in black (today) and cyan (LGM). The middle panel shows the Indian Ocean and the Pacific Ocean is shown in the right panel in a similar fashion. The percentile area during the LGM when compared to today for each western and eastern side of the ocean basins are shown.

Figure 3 shows the decrease of shelf areas between today and the LGM in different ocean basins. The shelf areas along the western coastline of the Atlantic Ocean decreased by 80% and by 76% along the eastern coastline, excluding the Mediterranean Sea. The shelf areas within the Mediterranean Sea did not change as much with about 40% remaining at the LGM. The biggest change happened in the Arctic Ocean with an immense decrease of shelf area due to the lower sea level and especially the greater extent of glacial and permanent sea ice (e.g. Fig. 1). Another highly affected area is the Atlantic coast of South America, where some of its shelf areas disappeared completely (Fig. 3).

Although the Indian Ocean does not have extensive shelf areas, those which do exist, were reduced by more than half. The western side from the tip of the Indian peninsula along the African coast were about 57% smaller than today. The area on the eastern side of the Indian Ocean along the Indonesian coast down to Australia was about 66% smaller than today.

The shelf area of the Pacific Ocean were also much smaller than today. Similar to the Atlantic Ocean, the biggest change happened in the Arctic Ocean, where most shelf areas were not accessible below the permanent ice cover (Fig. 3). The shelf area on the western side was approximately 70% smaller than today, and the quantitative reductions in actual square kilometres were immense.

Such decline in available shelf habitat must have had a profound effect on all organisms supported by these diverse and productive shelf seas today. To investigate such effect, we focus on one marine mammal species with limited diving capabilities, which uses the highly productive shelf areas for foraging.

### Current grey seal habitat

**Figure 4:**
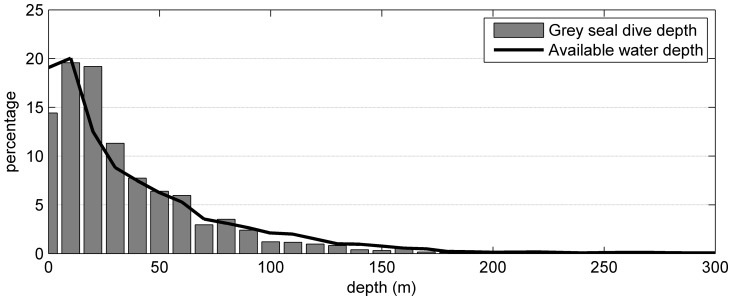
Dive depths of grey seals. Binned dive depths of grey seals based on 223,157 dives (grey bars) and available water depths at 34,140 locations (black line) from the ETOPO1 1 arc-minute global relief model.

To compare the current extent of the habitat available to grey seals with that available at the LGM, we needed to define their current habitat using very simple proxies, which are available to us for the LGM (water depth and surface temperature). We grouped the water depths extracted for each received seal location from the ETOPO1 dataset and the dive depths recorded by the SRDLs into 10 m bins (Fig. 4). The maximum dive depth recorded was 477 m. However, 95% of all recorded locations are associated with water depths of less than 127.5 m and 95% of all recorded maximum dive depths are less than 113 m. This agrees with results of previous studies that grey seal spend their time solely on the shelf (Fig. 2) and dive most of the time to the sea floor for foraging [37]–[39], [41], [42], [48]–[50].

**Figure 5:**
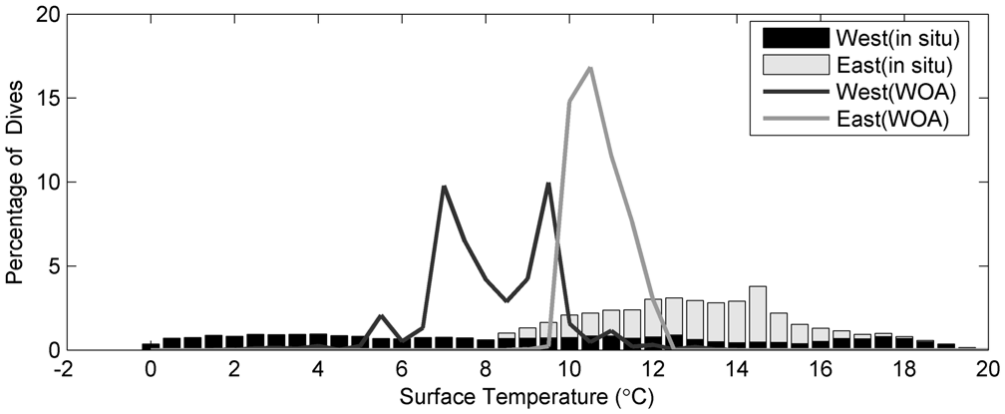
Annual mean surface water temperature experienced by grey seals. Binned surface water temperatures measured by SRDLs (bars) and calculated from the gridded SST data (lines) at the dive locations for grey seals east of 30°W (light coloured) and west of 30°W (dark coloured).

**Figure 6:**
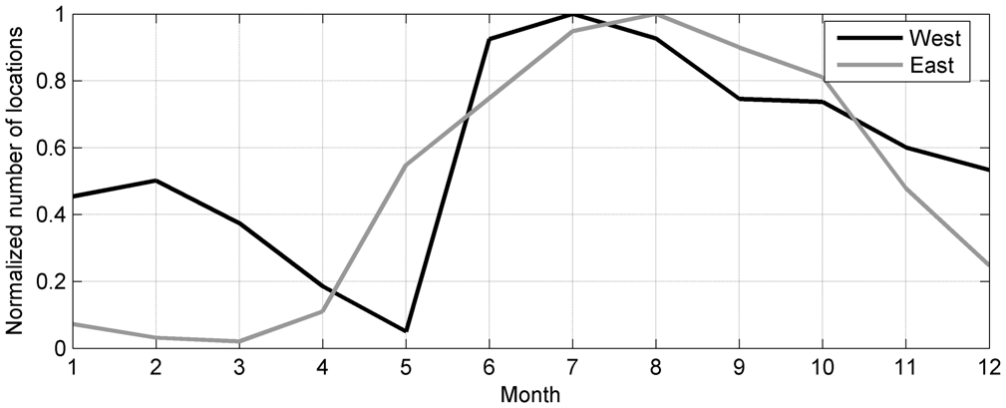
Seasonal distribution of telemetry data. Normalized number of received locations per month from SRDLs deployed on grey seals in the western (black) and eastern (grey) North Atlantic.

From each temperature-depth profile measured by SRDLs, we took the temperature reading closest to the surface (typically at 5–7 dbar pressure) and summed them into 1°C bins (Fig. 5) separately for the western and eastern population. The 95% water temperature range of 0°C to 20°C recorded from the western North Atlantic grey seal sample was wider than the range of 8.5°C to 18.5°C recorded from the eastern North Atlantic grey seals. However, these measurements also incorporate a temporal component. Seals in the eastern population were tagged after their moult in January to March and the SRDLs stopped working usually in late summer (Fig. 6). Therefore, the temperature data of the eastern population do not represent year around water temperatures. The moult of the western population is later and animals were mainly tagged in June (Fig. 6). Hence, the resulting temperature coverage obtained by SRDLs fitted to the western population better captures the minimum and maximum sea temperatures within a year. Combining the two populations results in a temperature range of 1°C to 17.5°C for 95% of measurements.

We also interpolated the annual quarter degree SST data of the World Ocean Database 2005 on the 34,140 SRDL locations. These SST data are also shown in figure 5. Again there is a distinction between the western and eastern population, with the western population experiencing colder waters. For the combined sample of all grey seal locations the annual sea surface temperatures range from 2.7°C to 12.6°C with 95% of all locations within 5.4°C to 11.7°C, which matches closely the results from the in-situ temperature range.

**Figure 7:**
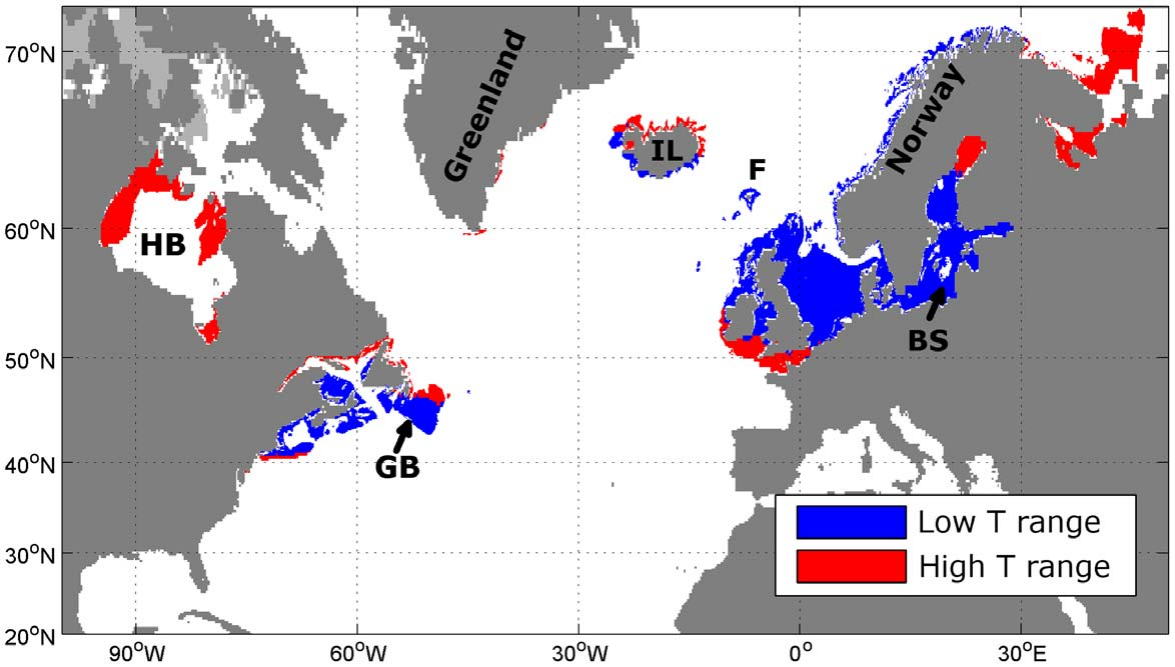
Today's grey seal range. Predicted grey seal habitat today based solely on water depth and annual climatological sea surface temperature data. For this estimate, grey seals inhabit a depth range between 0 m and 127.5 m and a SST range of 5.4°C to 11.7°C (blue) or 2.7°C to 12.6°C (blue and red). Isobath is 500 m, land is shaded dark grey and summer ice cover is light grey. Some important topographic features are marked: Hudson Bay (HB), Grand Banks (GB), Iceland (IL), the Baltic Sea (BS) and the Faroe Islands (F).

We then used these ranges extracted from the World Ocean Database to predict today's grey seal habitat (Fig. 7). We initially assumed that grey seal habitat is defined by a water depth of less than 127.5 m and a range of mean annual SST of 5.4°C to 11.7°C. We used the 5-minute elevation data [25] to retrieve grid cells within the water depth range. Then, for each of those grid cells we derived the annual mean SST from the gridded SST dataset. Grid cells in inland lakes, Lake Ladoga and the Caspian Sea were removed. We also removed grid cells with permanent summer ice cover (Fig. 1) and summed up the remaining grid cells. The total calculated area for the current grey seal habitat is then estimated to be 1.42·10^6^ km^2^. Extending the habitat SST range to 2.7°C to 12.6°C increases the estimated total area to 2.07·10^6^ km^2^ (Fig. 7).

### Paleoclimatic grey seal habitat

**Figure 8:**
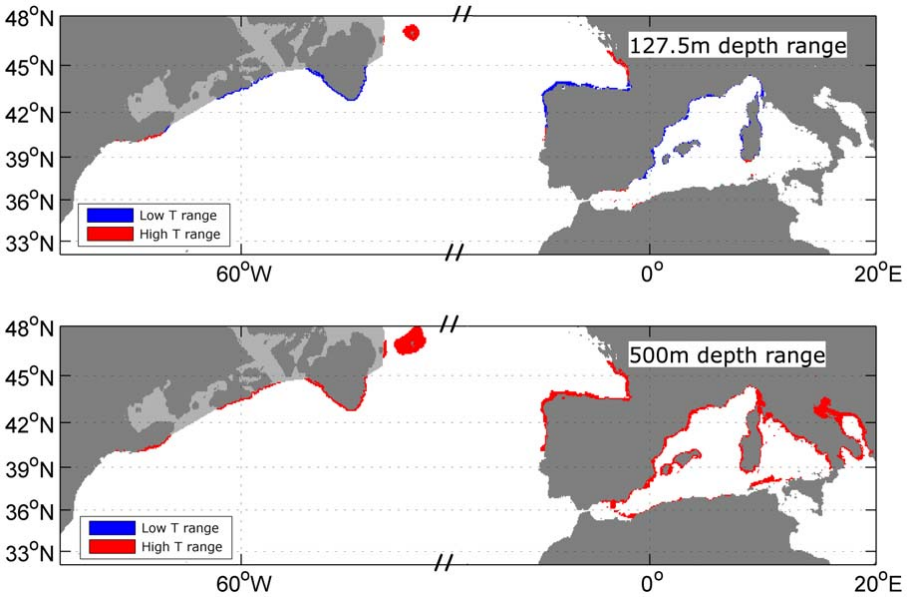
Grey seal range during LGM. Predicted grey seal habitat during LGM with drop in sea level of 123 m based solely on water depth and annual reconstructed sea surface temperature data. *Top*: For this estimate, grey seals inhabit a depth range between 0 m and 127.5 m and a SST range of 5.4°C to 11.7°C (blue) or 2.7°C to 12.6°C (blue and red). *Bottom*: For this estimate, grey seals inhabit a depth range between 0 m and 500 m and a SST range of 2.7°C to 12.6°C (red). Land is shaded dark grey and summer ice cover is light grey. Water depth contour is omitted for better clarity of the coloured areas.

We used the same depth and temperature proxies to define habitat extent at the LGM. Again, we assume that a water depth of less than 127.5 m and a range of annual mean SST of 5.4°C to 11.7°C define the grey seal habitat, but also used the 2.7°C to 12.6°C temperature range as extreme values. We then calculated the habitat areas using the GLAMAP SST climatology (Fig. 1 and 8). Assuming a sea level drop of 123 m between today and the LGM, the habitat is reduced to 4.74·10^4^ km^2^ for the smaller temperature range and 6.87·10^4^ km^2^ for the extreme SST range. These values correspond to a habitat loss of nearly 97%. Figure 8 shows the available habitat to the grey seals during the LGM. The previously large shelf areas were not available and the habitat was restricted to a narrow belt along the coastline. In the western North Atlantic the grey seal range was limited to the continental shelf edge between 40°N and 45°N and around Flemish Cap, which was an island during the LGM. On the eastern side, the coast of the Bay of Biscay and the north-eastern coast of the Iberian Peninsula would have been suitable. In contrast to today, the simple model also highlights the coasts of the western Mediterranean as suitable grey seal habitat during the LGM (Fig. 8).

## Discussion

### Prediction of today's habitat

The most exciting outcome of this study is the possibility of estimating the current grey seal habitat by two simple proxies (SST and water depth). These simple metrics chosen to describe grey seal foraging habitat were determined by the types of comparable information available from both the GLAMAP-2000 model and the satellite telemetry studies.

By using an annual mean SST range of 5.4°C to 11.7°C and a maximum water depth of 127.5 m we are able to predict today's grey seal habitat in accordance with tracking studies even in areas from which no telemetry data were used for this study (Fig. 7). Despite the use of a geographically restricted data set to derive temperature and depth ranges, the predicted foraging habitat range agrees with the current known world distribution of grey seals as published by the International Union for Conservation of Nature or the National Marine Fisheries Service. For example, the predicted range includes the Baltic and the Norwegian and Russian coasts for which no data were included in the sample. While the grey seal population in the Baltic Sea is quite low, our model classifies it at suitable habitat and indeed the Baltic area was known as an important seal oil producer in the last 2000 years until the numbers decreased dramatically in the 20^th^ century [51]. Grey seals are also known to populate the outer islands along the Norwegian coastline [52], [53], the Faroese waters [54] and the Icelandic coast [55]. The simple model also captures the present population in Maine, U.S. [56] and the past population along the northeast coast of the U.S. [57].

However, today's grey seal range cannot be sufficiently described using only the minimum SST range, as seals are found outside of this area as well. Haug et al. [52] describe a population living along the Murman coast in Russia and Rosing-Asvid [58] mentions that the first grey seals were observed in Greenland in 2009. Therefore, the use of the extended SST range for predictions today and during the LGM in this study is justified. Nevertheless, when using this extended SST range, grey seals do not use some apparently suitable habitat. The absence of seals on Grand Banks is apparent (Figs. 2 and 7), but in 2010 an adult male grey seal was tracked moving onto Grand Banks and spending time there (D. Bowen, unpublished data). Also, the waters in Hudson Bay may simply not be accessible from the current range (Fig. 7), but grey seals have been observed as far north as the northern tip of Labrador in the summer [59]. This could show that grey seals in the eastern Atlantic may still be expanding into habitats they previously occupied (before extensive hunting) or which open up due to reduced ice cover (Hudson Bay).

The southern-most limit of the eastern Atlantic population is not accurately predicted using the annual mean SST ranges based on the ARGOS locations. A small population of less than 200 individuals uses haul-out sites in Brittany, France [36], [40], [60], [61] which is further south than predicted. This sub-population inhabit water slightly warmer than the 95% range of mean SST but the average summer temperature is well within the 95% range of the in situ recorded SST values from the 81 seals in the sample used here. Interestingly, tracking studies have shown that grey seal tagged in Brittany generally forage to the north in waters off Ireland, Wales and the Channel Islands, areas captured by our simple model [61].

While this simple approach seems to define the habitat of tracked seals well, the definition of today's habitat based on just two simple proxies only indicates the overall range of grey seals, but does not give any indication of how this range is utilised. Much more sophisticated analytical techniques are needed to study habitat selection and species distribution [62].

The use of annual mean SST from the World Ocean Database 2005 to define suitable habitat appears to ignore the extremes as shown by the SRDL measurements (Fig. 5). There are two reasons for selecting this apparently less responsive temperature descriptor. Firstly, the temperature sensors were not calibrated before deployment and the accuracy is supposed to be within 0.5°C as post-deployment calibration is usually not possible. This error can result in values higher or lower than actually encountered. Secondly, and more importantly, the SST range is calculated from the annual climatology so that extreme values are averaged out. The measured high temperatures (Fig. 5) above 13°C are most likely warm water patches close to the shore in shallow water, which are not resolved in the climatology.

### The global loss of shelf areas

The lowering of sea level during glacial periods is well documented and its effects in exposing areas of shallow continental shelf to terrestrial animals are well known. For example, the central and southern North Sea and the Bering Sea land bridge are known to have been extensive and productive terrestrial habitats during and shortly after the LGM [63], [64]. We showed a general loss of shelf area (<500 m) of about 73%, when compared to today. However, the corresponding habitat loss and effect to the marine environment has still attracted little attention [17], [65]–[67]. Today, the primary production on the shelf is about 16% of the global ocean production [20], [21] and about 90% of global fish catches come from this area [68]. This biologically rich environment supports large populations of marine mammals and seabirds [22]. Consequently, any kind of shelf area loss is expected to have cascading effects on the food web on and off the shelf. For example, the shallow-water benthos must have been non-existent in high latitudes or at least very different [67] and the change in distributions of planktonic organisms have provided the basis of most LGM reconstructions [26]. But only recently have studies started to investigate the specific fate of more complex species over the last couple of oscillations of the ice-sheets [17], [67].

This habitat loss must have had profound effects on all marine mammals utilizing these areas, especially in the Arctic and high latitudes, where the habitat loss was even greater (>90%), but which are marine mammal hotspots today (Fig. 3). For the Arctic, it might be argued that ice breeding seal species were not affected by this and adapted by moving south with the ice, but most of them feed on shelf areas [69]–[71] and as a result must have dealt with habitat loss and a different ecosystem by either changing their behaviour or through reduction in numbers. This knowledge about the global loss of marine habitat and its impact on marine mammal populations together with the information available of paleoclimatic conditions needs to be exploited more to investigate population fluctuations on long time scales. This knowledge will enable us to address impending biological changes to these marine ecosystems. However, the effects on specific species need to be discussed elsewhere, while we focused on one specific example.

### Paleoclimatic grey seal habitat

The major loss of suitable foraging habitat as defined by our study must have had a major impact on the grey seal population size. At present there are around 300,000 grey seals over 1 year old in the entire North Atlantic [72]. There are indications that over much of their range grey seal populations are approaching carrying capacity with either stable populations or gradually declining rates of increase [72], [73]. However, we do know that the carrying capacity has not been reached in the Baltic Sea, the southern North Sea or the Northwest Atlantic and, in any case, it would be dangerous to assume that current carrying capacities are indicative of conditions before human perturbation of marine ecosystems. Hence, it is not possible with any confidence to estimate the natural maximum world population size for grey seals. However, if for illustration we speculate that the total number of grey seals (age 1 and older) could reach 500,000–700,000 we can estimate the LGM population assuming an even distribution of these seals across the possible habitat. We would then estimate the LGM population to have been around 15,000–21,000 seals. This would represent a very small global population for a phocid seal species, especially since it would have been split into two separate populations on either side of the North Atlantic. For comparison, this would represent a population smaller than the current estimates for any phocid species other than the critically endangered monk seals [74] and would have qualified as an endangered species under IUCN criteria [2], [75].

**Figure 9:**
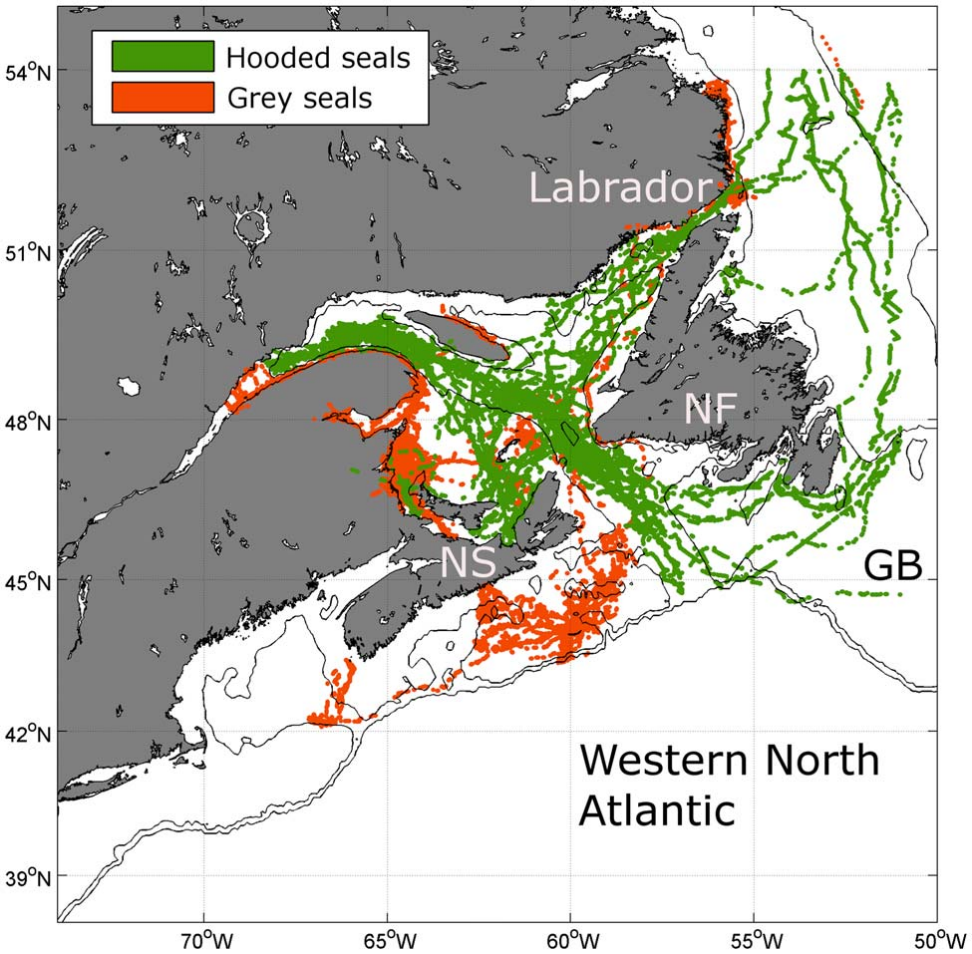
Grey and hooded seal telemetry locations. Grey seal locations (orange dots; subset of figure 2) and locations from instruments deployed on Hooded seals (green) in the Western North Atlantic showing the division of the shelf habitat between the two species. Isobaths are 150 m and 500 m and land is shaded grey. Some important topographic features are marked: Newfoundland (NF), Nova Scotia (NS) and Grand Banks (GB).

A larger population may have been possible if there was a major change in grey seal behaviour. The absence of shallow shelf water would have required a shift to a more pelagic or bathy-pelagic feeding strategy. Grey seals today appear to prefer shallow shelf (<200 m) areas crossing deep troughs or channels to other shallow areas only infrequently (Figs. 2 and 4). Only a few dives (<0.8%) deeper than 200 m were recorded within the western population, when most of the shallow shelf is covered by winter sea ice. In today's habitat, only hooded seals (*Cystophora cristata*), another large abundant pinniped that winters in these areas, use such shelf-slope habitat with water depths between 150 m and 500 m. In the western North Atlantic grey and hooded seals occur at the same latitude (seasonally) and do overlap in a few areas, in which only one depth range is available. However, within the Gulf of St. Lawrence satellite telemetry data from this region shows a remarkable distinction between grey and hooded seal distributions, with an apparent border coincident with the 200 m contour and little movement by either species across this boundary (Fig. 9).

There is no reason to suspect that the habitat preferences and or relative abilities to exploit shallow and deep shelf waters of grey and hooded seals would have been different at the LGM. However, if we make the extreme assumption that grey seals could have out-competed hooded seals during the LGM and we relax the water depth constraint to 500 m for the predicted paleoclimatic grey seal habitat (Fig. 8), the total calculated area increases to approximately 2.11·10^5^ km^2^, which is three times more than the extended area. This could then have potentially supported up to 63,000 seals in ideal conditions. However, this scenario seems unlikely given current seal distribution patterns.

Further extension of the suitable habitat to include deep ocean waters to forage does not seem reasonable as there is no evidence that grey seals feed over very deep water, regularly travel across ocean basins or go on extended foraging trips covering great distances. So, it seems likely that grey seal population fell to very low levels during the LGM and it would have remained low for several thousand years before expanding into current habitats over the last 12,000 years or so.

We have shown that we can describe current grey seal habitat using two simple proxies. This produces an accurate description of the effective range of grey seal populations based on observations and telemetry studies. Using these two proxies to define the extent of grey seal habitat during the LGM, indicates that it was only about 3% in size compared to today. We therefore conclude that the grey seal population during the LGM must have been very low for a considerable period of time.

In the future, Arctic permanent sea ice levels are predicted to get smaller and SST is predicted to increase in the Arctic [11]. For grey seals, this is likely to result in new available habitat and opportunities to extend their range onto the extensive Arctic shelf. However, grey seals will have to compete with other species either occupying this habitat currently or extending their range as well. It is also important to note that the apparent relationship between foraging habitat and some measures of water temperature is not likely to indicate direct physiological limits on grey seals. It is more likely that these relationships result from responses of their major prey items. Significant shifts in prey distributions in response to changing temperatures have already occurred in parts of the grey seal range [76]. Human impact and exploitation also need to be considered. At present we do not have sufficient information to allow us to predict the effects of these and future changes in prey distributions on grey seal foraging success.
